# Cost comparisons and factors related to cost per stay in intensive care units in Belgium

**DOI:** 10.1186/s12913-023-09926-2

**Published:** 2023-09-13

**Authors:** Arnaud Bruyneel, Lionel Larcin, Dimitri Martins, Julie Van Den Bulcke, Pol Leclercq, Magali Pirson

**Affiliations:** 1 Health Economics, Hospital Management and Nursing Research Dept, School of Public Health, Université Libre de Bruxelles, Brussels, Belgium; 2 Research Centre for Epidemiology, Biostatistics and Clinical Research, School of Public Health, Université Libre de Bruxelles, Brussels, Belgium

**Keywords:** Cost, Intensive care unit, Health care economics

## Abstract

**Background:**

Given the variability of intensive care unit (ICU) costs in different countries and the importance of this information for guiding clinicians to effective treatment and to the organisation of ICUs at the national level, it is of value to gather data on this topic for analysis at the national level in Belgium. The objectives of the study were to assess the total cost of ICUs and the factors that influence the cost of ICUs in hospitals in Belgium.

**Methods:**

This was a retrospective cohort study using data collected from the ICUs of 17 Belgian hospitals from January 01 to December 31, 2018. A total of 18,235 adult ICU stays were included in the study. The data set was a compilation of inpatient information from analytical cost accounting of hospitals, medical discharge summaries, and length of stay data. The costs were evaluated as the expenses related to the management of hospital stays from the hospital’s point of view. The cost from the hospital perspective was calculated using a cost accounting analytical methodology in full costing. We used multivariate linear regression to evaluate factors associated with total ICU cost per stay. The ICU cost was log-transformed before regression and geometric mean ratios (GMRs) were estimated for each factor.

**Results:**

The proportion of ICU beds to ward beds was a median [p25-p75] of 4.7% [4.4–5.9]. The proportion of indirect costs to total costs in the ICU was 12.1% [11.4–13.3]. The cost of nurses represented 57.2% [55.4–62.2] of direct costs and this was 15.9% [12.0-18.2] of the cost of nurses in the whole hospital. The median cost per stay was €4,267 [2,050–9,658] and was €2,160 [1,545–3,221] per ICU day. The main factors associated with higher cost per stay in ICU were Charlson score, mechanical ventilation, ECMO, continuous hemofiltration, length of stay, readmission, ICU mortality, hospitalisation in an academic hospital, and diagnosis of coma/convulsions or intoxication.

**Conclusions:**

This study demonstrated that, despite the small proportion of ICU beds in relation to all services, the ICU represented a significant cost to the hospital. In addition, this study confirms that nursing staff represent a significant proportion of the direct costs of the ICU. Finally, the total cost per stay was also important but highly variable depending on the medical factors identified in our results.

**Supplementary Information:**

The online version contains supplementary material available at 10.1186/s12913-023-09926-2.

## Background

Intensive care unit (ICU) costs are a significant part of total hospital costs, representing 8%-30% of the total hospital expenditures and ICU beds account for approximately 10%-30% of all hospital beds [1–3]. According a systematic review, ICU cost per day vary between 200 € to 4,322 € [2]. This large variability in ICU costs can be explained by several factors. Firstly, some of these studies are old and the costs vary greatly depending on the organisation of ICUs in the country [4]. Indeed, ICUs in Belgium are mostly mixed units (surgical and medical), non-sectoral, with no differences in care level, and general intermediate care (except stroke units and coronary units) does not exist [5]. To our knowledge, no study on cost analysis has been conducted in Belgium and it would be interesting to conduct a study with this particular ICU organisation. Secondly, the analysis of ICU cost data can be affected by many factors, including the diversity of study designs, case mix, costing methodologies, and the predictive power of the models used. Indeed, according to a systematic review, a wide range of costs per day is observed because there are two main costing methodologies for determining ICU costs: ‘‘top-down” and ‘‘bottom-up” cost analysis [2]. Top-down costing methodology calculates the average cost per patient or per diagnosis related group (DRG) [6, 7]. This method requires data at the department level, is straightforward, and can support budgetary decisions at political and hospital level. Its disadvantages are that it is less precise, can only be used for retrospective assessment and cannot be used for cost evaluation of subpopulations, particularly in the ICU [3]. Conversely, the bottom-up costing methodology calculates the actual cost per individual patient or subpopulation and is the best method for most economic evaluations. Furthermore, it enables statistical analyses and is more precise. On the other hand, this method is more lengthy and expensive, as it requires data at the patient level and activity level [8]. Multi-centre bottom-up studies are considered to be the most well-designed studies, providing more precise economic information that is valuable for cost-effective decisions. Thirdly, there are several types of cost which may influence interpretation of total cost. ICU costs can be direct or indirect. Direct costs usually cover over 80% of total ICU costs, with the salaries of nursing and medical staff representing the highest proportion of the fixed costs [2, 9, 10]. Indeed, the cost of caregivers is the most important cost factor in the ICU [1, 6, 11]. It is estimated that about half of the total costs in the ICU are spent on nursing staff [12–14]. However, this cost is influenced by the nurse-to-patient (N:P) ratio, the level of remuneration, and nurse education level, and these are not uniform in different countries [15–19]. An analysis of the cost of health care personnel and its proportion of direct costs is, therefore, interesting to carry out at the national level. Fourthly, this information is important because health care costs are constantly increasing. It is, therefore, essential that more studies focus on calculating the real costs of intensive care within the health care system [12]. Estimating the real costs and identifying the factors associated with the total costs of intensive care will help health care staff to provide more effective and, at the same time, possibly less expensive treatment. Finally, well-organised studies will help health policy makers to take the right decisions for example in financing, to make comparisons between profiles of patients and hospitals, and to achieve cost-effective management [20].

Given the variability of ICU costs in different countries and the importance of this information, it is relevant for healthcare decision making to carry out a study on this subject in Belgium. The objectives of this study were to describe the cost of ICUs and the factors that influence them the ICU in Belgium.

## Methods

### Patients and setting

This was a retrospective cohort study using data for the intensive care units of 17 Belgian hospitals from January 01 to December 31, 2018. These 17 hospitals represent 18.84% of hospital stays in Belgium. A total of 3,173 patients were excluded from the analysis, including 757 paediatric patients (< 16 years), 1,561 incomplete stays (no administrative data found in the hospital, very short ICU stay < 6 h, no data on costs per pathology, etc.), and 855 patients still hospitalized on December 31, 2018. A total of 18,235 adult ICU stays were included in the study.

### Context of the study in Belgium

In Belgium, the legal N:P ratio is 1:3 with wide heterogeneity between hospitals [21, 22]. Logistics assistants, physiotherapists, and care assistants are present in a majority of ICUs, but only usually during the morning shift, and ICU nurses generally work in three shifts. Concerning the training level of the nurses in the study, nurses had one of two levels of training (bachelor’s degree or no bachelor’s degree), as well as a specialisation which takes place after completion of the bachelor’s degree with an additional year that includes training in intensive and emergency care (approximately 80% of nurses) [22].

Hospital funding is mixed in Belgium. A first part of hospital funding is financed by the budget of financial means (37.3%) which is linked to the activity of the hospital and evaluated by the diagnosis-related groups (DRG), and the remainder of the funding comes from medical procedures (39.8%), pharmaceutical products (18.4%), and conventions (4.5%) [23]. The financing of nursing care, including that of intensive care nurses, comes from part of the budget of financial means and is adjusted according to the nursing activity Minimum Hospital Dataset [24, 25]. Academic hospitals have additional funding for research and teaching. The funding of intensive care is complex and is not based on real ICU capacity. A calculation of a bed calculation beds with intensive care characteristics based on the overall activity of the hospital: a list of extensive medical services (20%), the intensive care nursing profile (40%), and the national percentage of intensive care by APR-DRG (40%). It should be noted that a hospital may, therefore, have more or less actual ICU beds than financed beds. These beds have more nursing staff than beds in the wards.

### Data collection

The data set was a compilation of inpatient information from, medical discharge summaries, length of stays (LOS) in 2018 and cost analytical cost accounting.

The costs in this study refer to expenses for the acute management of hospital stays from the hospital perspective. The cost from the hospital perspective is calculated using a cost accounting analytical methodology in full costing [23, 26]. The costs of ICU care have been subtracted from the total cost of the stay. A complete cost per hospital stay was calculated from the hospital perspective. The hospital cost took into account the direct and the indirect costs. Direct costs were costs related to patients during their stay in the ICU and indirect costs are costs that cannot be directly attributed to patients within the ICU (Appendix 1). All amounts in the study are expressed in euro (€). The occupancy rate was obtained through the number of stays and LOS in minutes adapted to the number of ICU beds for a year. The number of full-time equivalents (FTEs) was obtained from the accounting data of the hospital. The nursing costs for the hospital were obtained via the cost accounting of hospitals.

The allocation of nursing care costs in the ICU was made according to two criteria. The first one was based on the OMEGA scale, which uses some medical procedures and care performed to patients [27]. This scale, created in France and composed of 47 items, was adapted to this study for activities usually recorded in the ICU. 21 items (40 Belgian activities) and 4 drug classes (12 ATC [Anatomical Therapeutic Chemical] codes) have been considered. Originally, the Omega scale was not intended to assess nursing workload but to allocate hospital costs. The second criterion is the LOS in the ICU. According to a consensus of experts, a 50/50 ratio between these two criteria was used for the nursing care cost allocation.

The main diagnostic and sociodemographic data (e.g., age, sex, mortality) were obtained via the minimum hospital discharge summary and administrative data. The Charlson score was also calculated through the minimum hospital discharge summary data system with International Classification of Diseases-10 and administrative data [28]. The scale was developed in 1984, so the authors re-evaluated the Charlson index in 2011 and re-assigned weights for each comorbidity by tracking mortality in the year following hospital discharge. The updated index and weights were applied to hospital discharge data from six countries and tested for their ability to predict hospital mortality [29]. This score showed good agreement and predicted 30-day and 1-year mortality in ICU patients [30, 31]. Medical procedures (e.g., mechanical ventilation and duration, extracorporeal membrane oxygenation (ECMO), continuous hemofiltration) were obtained through invoicing files.

### Statistical analysis

Statistical analyses were performed using the statistical software STATA® version 15. A p-value < 0.05 was considered statistically significant. Characteristics of ICU patient stays are presented as proportions for categorical variables and means with standard deviations (SD) for quantitative variables or medians with interquartile range (IQR) for asymmetric variables. Univariate and multivariate linear regressions were performed to identify factors associated with hospital costs in the ICU. Linear regression models were performed on log-transformed hospital costs, to normalize the distribution of residuals. Additionally, the LOS was categorized into quartiles. When the assumption of homoscedasticity was not met, robust standard errors were computed to control for heteroscedasticity. In the univariate analysis, the raw geometric means of hospital costs and the coefficients from linear regression were computed for each indicator. We considered in the multivariate model the factors with a p value below 0.05 in the univariate analysis. To appreciate the adjusted effect of each predictor on the dependent variable in the multivariate model, we exponentiated the coefficients to obtain the adjusted ratio of geometric mean (GMR). For example, a specific category with a GMR of 1.25 was interpreted as a hospital cost 25% greater compared to the reference group.

The inpatient records used in the retrospective study were fully anonymised by the hospitals and the research team did not have any access to medical files.

## Results

Sociodemographic characteristics for the ICU patient stays included in the study are shown in Table 1. Mean (± standard deviation) age was 63.1 ± 16.8 years and proportion of geriatric patients was 24.4%. The median [p25 - p75] Charlson score was 4.4 [2.3–7.4], the proportion of ventilated patients was 30.4%, and the duration of mechanical ventilation was 4 [2–8] days. The most common main diagnosis was post-operative monitoring who are patients admitted for monitoring minor surgical procedures (17.0%), followed by coma/convulsion (16.9%), and cardiogenic shock/cardiac decompensation (16.7%) (Table 1).

**Table 1:** Sociodemographic and medical characteristics of the included ICU stays

Characteristics	Total (n = 18 235)
Age, years, mean ± SD	63.1 ± 16.8
Geriatric cases (> 75 years), n (%)	4,386 (24.8)
Men, n (%)	10,663 (58.5)
Charlson score, median [IQR]	4.4 [2.3–7.4]
Ventilated patients, n (%)	5,552 (30.4)
Mechanical ventilation time in days, median [IQR]	4 [2–8]
Measurement of intracranial pressure, n (%)	197 (1.0)
ECMO, n (%)	93 (0.5)
Continuous hemofiltration, n (%)	753 (4.1)
ICU LOS in days, median [IQR]	1.8 [0.8–3.7]
Hospital LOS in days, median [IQR]	9.3 [4.8–17.6]
ICU Readmission, n (%)	1,136 (6.2)
ICU mortality, n (%)	1,882 (10.3)
Hospital mortality, n (%)	2,432 (13.3)
**Main diagnosis, n (%)**	
Sepsis/septic shock	1,323 (7.3)
Cardiogenic shock/cardiac decompensation	3,038 (16.7)
Decompensation of chronic respiratory failure	991 (5.4)
Coma/convulsion	3,088 (16.9)
Intoxication	474 (2.6)
Heart surgery	2,651 (14.6)
Digestive surgery	2,916 (16.0)
Post-operative monitoring for minor surgical procedures	3,110 (17.0)
Other	644 (3.5)

**Legend**: SD = standard deviation; ICU = intensive care unit; LOS = length of stay; ECMO = extracorporeal membrane oxygenation; IQR: interquartile range

**Fig. 1:**
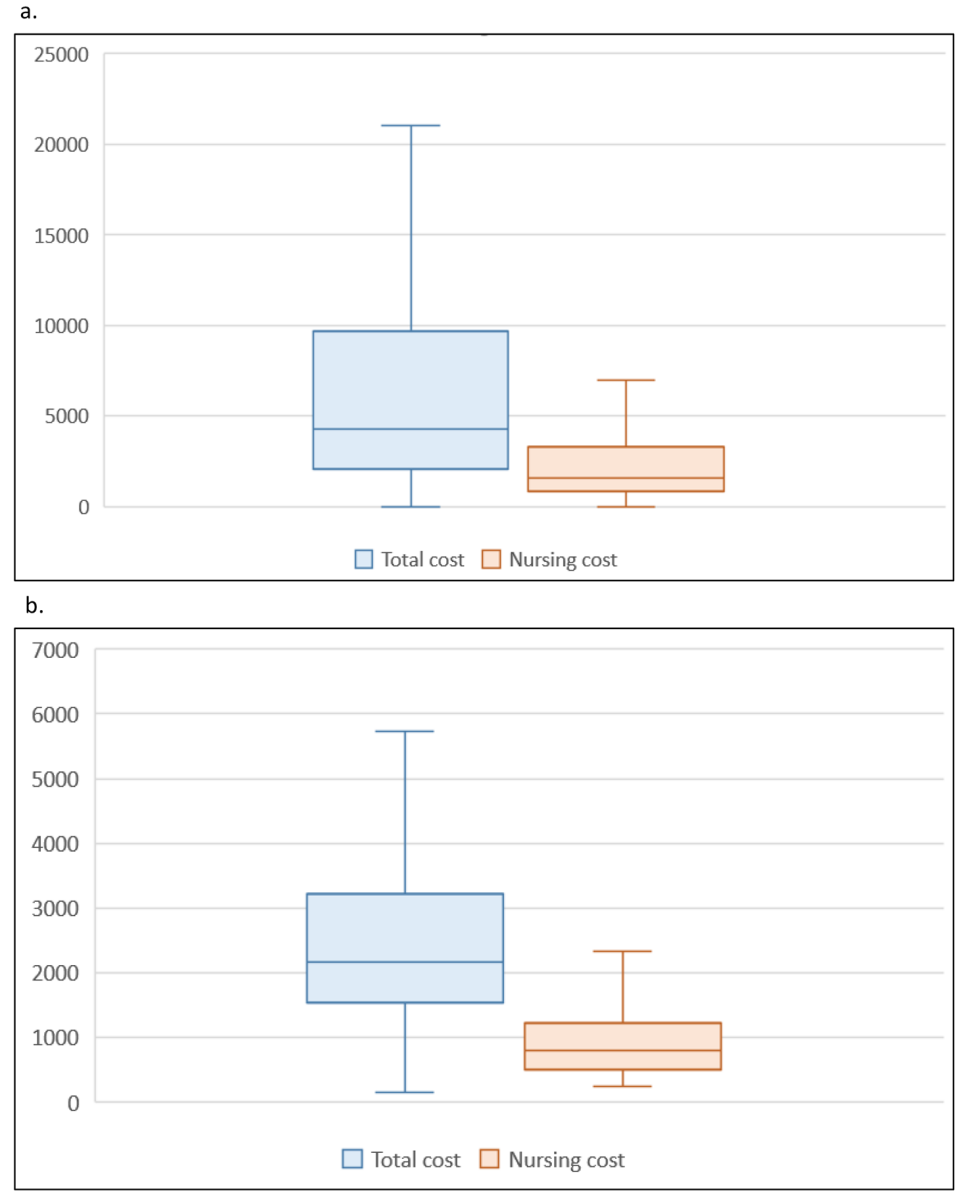
Box plots representing the total cost and the nursing cost per stay **(a)** and per day **(b)** in ICU (€)

Seventeen hospitals were involved in the study, including three academic hospitals, and the occupancy rate was 76.8% [69.5–83.3]. The proportion of ICU beds to inpatient beds was 4.7% [4.4–5.9]. The proportion of indirect costs in the ICU was 12.1% [11.4–13.3]. The nursing cost represented 57.2% [55.4–62.2] of the direct costs in the ICU, and ICU nursing costs were 15.9% [12.0–18.2] of the total nursing costs in the hospital. The median annual cost of a nurse full-time employee (FTE) was €75,593 [70,641–75,353] and the number of FTEs per ICU bed was 2.50 [2.10–2.80]. High variability in these results by hospital was observed. For the proportion of nursing costs to direct costs, one hospital (H12) was only at 35.4% because medical costs were more important in this institution. Conversely, the academic hospitals had higher nursing costs as well as costs per FTE. There was also variability in the median cost per stay per hospital, which was €4,454 [3,217–5,815] (Table 2). The median total and nursing costs per stay were €4,267 [2,050–9,658] and €1,574 [815–3,279], respectively. The cost per ICU day was €2,160 [1,545–3,221] and nursing cost was €789 [496–1,229] (Fig. 1).

**Table 2:** Description of hospitals and costs

Hospitals (n = 17)	University hospital	Occupancy rate ICU (%)	Proportion of ICU beds to ward beds (%)	Proportion of indirect costs ICU on the total cost (%)	Proportion of nursing costs to direct costs ICU (%)	Nurse cost ICU/ward nurse cost (%)	FTE nurse per ICU bed	Cost of one FTE nurse (€)	Median cost per stay per hospital (€)
1	No	74.0	3.0	13.0	55.0	12.0	2.07	75,968	3,003
2	No	76.8	6.0	12.6	56.7	18.4	2.17	74,630	6,112
3	No	62.3	5.7	11.4	59.5	18.2	1.71	74,373	4,595
4	No	82.6	4.7	13.4	47.2	17.8	2.81	72,983	8,641
5	No	68.7	3.4	13.3	57.2	13.6	2.48	67,932	4,903
6	Yes	76.1	4.4	8.2	57.9	12.7	2.69	79,429	3,429
7	Yes	85.8	7.3	11.4	65.3	28.8	2.92	82,251	3,996
8	No	87.0	5.0	15.5	56.3	23.2	2.97	77,352	5,793
9	No	71.7	4.9	15.7	59.9	17.5	2.19	75,593	5,884
10	No	78.0	6.5	9.0	62.2	23.1	2.89	76,113	5,251
11	No	89.9	2.7	17.8	65.4	11.8	2.38	82,101	4,454
12	No	98.1	3.7	11.9	35.4	15.9	2.48	70,641	7,376
13	No	66.8	5.9	12.1	51.2	16.8	2.56	70,326	3,217
14	No	69.0	4.5	13.1	71.4	11.4	2.60	76,234	3,216
15	Yes	82.0	4.5	10.8	68.4	12.1	3.21	81,273	3,077
16	No	82.1	4.5	10.6	55.4	14.1	1.90	70,054	3,490
17	No	69.5	3.4	11.9	56.4	8.2	2.19	67,555	2,466
Median [IQR] or n (%)	3 (18)	76.8 [69.5–83.3]	4.6 [3.7–5.7]	12.1 [11.4–13.3]	57.2 [55.4–62.2]	15.9 [12.0-18.2]	2.50 [2.10–2.80]	75,593 [70,641 − 77,353]	4,454 [3,217 -5,815]

ICU = intensive care unit; IQR = interquartile range; FTE = full time equivalent; n = absolute frequency; %=relative frequency.

In the univariate analysis, patients with ECMO have the highest geometric mean total costs (37,859, 95%CI: 31,946 to 44,865) followed by continuous hemofiltration patients (23,624, 95%CI: 22,064 to 25,294) and a Charlson score of 6+ (5,519, 95%IC: 5,1379 to 5,562).

In the multiple linear regression model, the main factors associated with higher cost per stay were Charlson score (GMR: 1.19 95%CI: 1.15 to 1.23 for score 6+), mechanical ventilation (GMR: 1.74, 95%CI = 1.63 to 1.86), ECMO (GMR: 1.88, 95%CI = 1.63 to 2.17), continuous hemofiltration (GMR: 1.62, 95%CI: 1.54 to 1.70), readmission (GMR: 1.17, 95%CI: 1.12 to 1.21), ICU mortality (GMR: 1.39, 95%CI: 1.34 to 1.43), academic hospitals (GMR: 1.15, 95%CI: 1.13 to 1.18), and diagnoses of coma/convulsions (GMR: 1.4, 95%CI: 1.10 to 1.18) and intoxication (GMR: 1.06, 95%CI: 1.02 to 1.10) compared to cardiogenic shock/cardiac decompensation. Conversely, heart (GMR: 0.93, 95%CI: 0.88 to 0.98) and digestive surgery (GMR: 0.91, 95%CI: 0.86 to 0.96) were associated with lower costs in the multivariate analysis compared to cardiogenic shock/cardiac decompensation. For LOS, each quartile was associated with an increase in cost. Compared with the first quartile of length of stay, the 3rd quartile involved an augmentation in cost of more than 300% (GMR: 3.366, 95%CI: 3.363 to 3.571) and the 4th quartile of LOS involved more than 800% augmentation in total cost − 832% (GMR: 8.332, 95%CI: 8.065 to 8.608) (Table 3).

**Table 3:** Factors related to total cost per ICU stay by univariate and multivariate analysis

	Univariate linear regression*		Multivariate linear regression **		
Factors	Total cost (€)	Not Adjusted	Adjusted	Adjusted	p value
	Geometric Mean (95%CI)	coefficient (95%IC)	coefficient (95%IC)	GMR** (95%CI)	
**Geriatric cases**					
Not geriatrics case	4,453 (4,365 to 4,543)	Ref	Ref	Ref	
Geriatrics case (> 75 years)	4,798 (4,639 to 4,963)	0.07 (0.03 to 0.11)	-0.05 (-0.07 to -0.02)	0.95 (0.93 to 0.97)	< 0.0001
**Gender**					
Men	4,767 (4,661 to 4,875)	Ref	Ref	Ref	
Women	4,225 (4,114 to 4,340)	-0.12 (-0.15 to -0.09)	-0.02 (-0.04 to 0.00)	0.99 (0.96 to 1.00)	0.068
**Charlson score**					
0 (n = 2 741)	2,938 (2,807 to 3,077)	Ref	Ref	Ref	
1–3 (n = 3 367)	4,061 (3,907 to 4,221)	0.32 (0.26 to 0.38)	0.12 (0.08 to 0.15)	1.13 (1.09 to 1.17)	< 0.0001
4–5 (n = 4 319)	4,560 (4,404 to 4,720)	0.44 (0.38 to 0.45)	0.12 (0.09 to 0.16)	1.13 (1.09 to 1.17)	
6+ (n = 7 806)	5,519 (5,379 to 5,662)	0.63 (0.58 to 0.68)	0.17 (0.14 to 0.20)	1.19 (1.15 to 1.23)	
**Mechanical ventilation**					
Not ventilated patients	2,972 (2,920 to 3,024)	Ref	Ref	Ref	
Ventilated patients	11,899 (11,598 to 12,209)	1.39 (1.36 to 1.43)	0.55 (0.53 to 0.58)	1.74 (1.63 to 1.86)	< 0.0001
**ECMO**					
Patients without ECMO	4,485 (4,409 to 4,562)	Ref	Ref	Ref	
Patients with ECMO	37,859 (31,946 to 44,865)	2.13 (1.95 to 2.30)	0.63 (0.45 to 0.77)	1.88 (1.63 to 2.17)	< 0.0001
**Continuous hemofiltration**					
Patients without continuous hemofiltration	4,223 (4,152 to 4,295)	Ref	Ref	Ref	
Patient with continuous hemofiltration	23,624 (22,064 to 25,294)	1.72 (1.64 to 1.79)	0.40 (0.43 to 0.53)	1.62 (1.54 to 1.70)	< 0.0001
**ICU LOS in quartiles**					< 0.0001
Quartile 1 [0–1] (n = 4,562)	1,454 (1,416 to 1,494)	Ref	Ref	Ref	
Quartile 2 [1-2] (n = 4,551)	2,930 (2,875 to 2,987)	0.70 (0.67 to 0.73)	0.67 (0.64 to 0.70)	1.96 (1.91 to 2.02)	
Quartile 3 [2-4] (n = 4,562)	5,593 (5,499 to 5,688)	1.35 (1.32 to 1.37)	1.24 (1.21 to 1.27)	3.47 (3.36 to 3.57)	
Quartile 4 [4-max] (n = 4,558)	17,726 (17,331 to 18,131)	2.500 (2.47 to 2.53)	2.12 (2.09 to 2.15)	8.33 (8.06 to 8.61)	
**Readmission ICU**					
Patients not readmitted	4,218 (4,145 to 4,269)	Ref	Ref	Ref	
Patients readmitted	13,448 (12,612 to 14,340)	1.16 (1.09 to 1.23)	0.15 (0.115 to 0.19)	1.17 (1.12 to 1.21)	< 0.0001
**ICU mortality**					
Surviving patients	3,954 (3,885 to 4,024)	Ref	Ref	Ref	
Deceased patients	10,795 (10,316 to 11,295)	1.00 (0.96 to 1.05)	0.33 (0.30 to 0.36)	1.39 (1.34 to 1.43)	< 0.0001
**Hospital academic**					
Inpatients non-academic hospital	4,659 (4,563 to 4,758)	Ref	Ref	Ref	
Inpatients academic hospital	4,274 (4,147 to 4,405)	− 0.09 (-0.13 to -0.50)	0.14 (0.12 to 0.16)	1.15 (1.13 to 1.18)	< 0.0001
**Main diagnosis**					
Cardiogenic shock/ cardiac decompensation	3,254 (3,124 to 3,390)	Ref	Ref	Ref	< 0.0001
Coma/convulsion	4,218 (4,062 to 4,380)	0.26 (0.20 to 0.32)	0.13 (0.09 to 0.16)	1.14 (1.10 to 1.18)	
Decompensation of chronic respiratory failure	6,821 (6,385 to 7,287)	0.74 (0.67 to 0.82)	-0.039 (-0.09 to 0.01)	0.96 (0.92 to 1.00)	
Sepsis/sepsis shock	6,514 (6,122 to 6,932)	0.20 (0.14 to 0.26)	-0.01 (-0.04 to 0.03)	0.99 (0.96 to 1.03)	
Intoxication	3,701 (3,399 to 4,030)	0.76 (0.70 to 0.82)	0.06 (0.02 to 0.09)	1.06 (1.02 to 1.10)	
Heart surgery	6,978 (6,687 to 7,281)	0.13 (0.02 to 0.24)	-0.07 (-0.13 to -0.00)	0.93 (0.89 to 0.98)	
Digestive surgery	3,978 (3,803 to 4,161)	0.03 (-0.07 to 0.13)	-0.01 (-0.15 to -0.04)	0.91 (0.86 to 0.96)	
Post-operative monitoring	4,355 (4,167 to 4,552)	0.29 (0.23 to 0.35)	0.00 (-0.03 to 0.03)	1.00 (0.97 to 1.04)	
Other	3,351 (3,077 to 3,650)	0.69 (0.62 to 0.77)	0.05 (0.01 to 0.09)	1.05 (0.976 to 1.10)	

**Legend**: ICU = Intensive care Unit; LOS: Length of stay; ECMO = Extracorporeal membrane oxygenation; IQR: Interquartile range.

*All variables were statistically significantly associated with higher total cost in the univariate analysis.

**Multivariate model includes as dependent variable: total cost and as independent variables: geriatrics cases, gender, Charlson score, mechanical ventilation, ECMO, continuous hemofiltration, ICU LOS in quartiles, readmission ICU, ICU mortality, hospital academic, main diagnosis. Adjusted R²=0.697 n = 18,233.

***GMR (exponential of coefficient): Geometric Mean Ratio is the ratio of expected geometric mean in the specific category to expected geometric mean in the reference category.

## Discussion

The aim of this study was to describe ICU costs and to analyse the factors associated with cost per ICU stay. Based on the results of this study, we can make the following observations.

First, the proportion of ICU beds in Belgian hospitals compared to total hospital beds is lower than in other European countries [32, 33]. However, Belgium has a higher number of ICU beds per inhabitant (on average 15.9 per 100,000 inhabitants) than the rest of Europe (11.5 per 100,000 inhabitants), because the number of beds in conventional units per inhabitant is also very high, which explains this relatively low frequency of ICU beds [33, 34]. There is variability in these results (from 2.7 to 7.3%) which may be due to intensive care activities (i.e., cardiac surgery, neurosurgery, oncological activity) but also to hospital activities (i.e., maternity, surgery, oncology). Given the reduction in LOS and the shift to day surgery and ambulatory medicine, it is likely that hospital beds will decrease in Belgium, while the number of ICU beds will theoretically remain constant. This proportion of ICU beds will certainly increase in the coming years [35].

Second, in this study, the proportion of direct ICU costs in relation to total hospital costs was 17.4% [14.1–19.7]. Compared to a study in the USA, Germany, and the Netherlands, ICU costs were around 20%, which is very similar to our results [12–14]. However, the comparison of this result is difficult as it may vary depending on the number of ICU beds in the hospital, the case mix, the methodology of the cost analyses, and the distribution of direct/indirect costs [6]. For Belgium, given the proportionally small number of ICU beds in relation to the total number of hospital beds, direct ICU costs are high. The proportion of indirect costs (12.1% [11.4–13.3]) in this study is also lower than that reported in the literature (usually 20%) but with the same limitations mentioned above for the comparison of costs with other studies [2].

Third, regarding the impact of nursing costs on direct costs, the results of this study are higher than the results of other European studies on the subject [3, 4, 9, 36]. However, this figure varies according to nurse salaries and the N:P ratio of the ICU, education level also may affect the reliability of this figure. [6]. When looking at the number FTEs per bed, the N:P ratio (about 1:2.5) is rather low but the impact of salary appears to be more important when looking at the cost of an FTE nurse [7, 18, 21, 37]. The cost of nursing staff thus represents a large part of the direct costs in Belgian ICUs. However, nursing staff can be considered as an investment as the costs avoided through reduced readmissions and shorter length of stay have been demonstrated with the provision of additional nursing staff [38–40].

Fourth, for the total cost per day, as in other studies, there was significant variability in the cost per ICU stay per day in this study which can be explained by the factors analysed in the study, such as patient comorbidities, mechanical ventilation, continuous hemofiltration, LOS, mortality, readmission, and type of diagnosis. The factors observed in the study are very similar to other studies on the subject despite the fact that cost analysis methods and ICU organisations may be very different. The multivariate analysis showed that patients hospitalised in academic hospitals had a higher cost per stay. This can be explained by the more severe pathologies and some confounding factors in academic hospitals. Conversely, some major pathologies (cardiac and digestive surgery) were less costly only in the multivariate analysis because of confounding factors. These observations can guide political authorities and hospital managers to hospital funding and to better manage health care facilities. In contrast, the median total cost per stay in this study was lower than in other studies. This may be explained by a relatively low proportion of ventilated patients and mortality and a low median LOS in the ICU. Looking at the factors associated with high total costs, the cost of ventilated patients is very similar to what is observed in the systematic review [2]. The cost of nursing care per ICU day and per stay is difficult to compare. Indeed, it depends on the methodology of allocating nursing costs per patient and the N:P ratio [7, 41]. However, a recent study showed a strong correlation between our nursing cost allocation methodology and the Nursing Activities Score (NAS), which is the instrument of choice for nursing cost allocation [24, 42].

Finally, regarding the case-mix in this study, age, diagnosis, and mortality rate in the ICU were similar to previous studies in Belgium [21, 43]. The mortality rate, proportion of patients ventilated, and median LOS were low compared to other European studies [44]. The overall ICU readmission rate (6.2%) was lower than that previously reported in the literature, approximately 10% in the same hospitalisation [45, 46]. What is more surprising is the proportion of patients admitted for post-operative monitoring (17%). Belgium does not have different levels of intensive care units, which results in less severe admissions and cases than in other European countries, the creation of an intermediate care unit and/or a postoperative surveillance unit could reduce admissions and LOS for ICU patients [47–50]. In addition, there is also a high degree of variability in the analysis of costs per hospital in our sample (e.g., the cost of nurses in relation to direct costs or the number of FTE nurses per ICU bed and those costs). It seems that even within the same country, ICU activity and investment in nurses are not identical. This can be explained by more specific ICUs (e.g., cardiac surgery or neurosurgery) and, therefore, different nursing activities [21]. An assessment of the workload also seems appropriate to adapt nursing resources to the needs of the patients [24].

For perspective, this study can also be used to provide reference costs for other medico-economic, cost-effectiveness studies and budgetary impact studies in the ICU [51]. The financing of ICUs, based on DRG, as in most European countries [11], is also questionable due to the variability of the total costs per stay and per day. We have identified factors that influence the cost of the stay and these could be used to fund according to ICU activity.

## Limitations

This study has certain limitations. Firstly, the variables included in the model were used retrospectively and not prospectively extracted from hospital databases. Patient variables are derived from billing data and do not always reflect what is actually provided to the patient. Secondly, the cost calculations should be compared carefully, as the methodology used to calculate the cost and the perspective may differ. Thirdly, anonymised administrative data do not allow us to collect the medical severity of the patient, which is also a risk factor and cost predictor for the hospital. However, pathology and some invasive treatments (i.e., mechanical ventilation, ECMO, haemofiltration) and comorbidities (Charlson score) were included in the multivariate model. Fourthly, the analysis of the cost of nursing staff partly using an Omega scale that has not been internationally validated. However, this breakdown of nursing costs seems to have performed well compared to international scales [24]. Finally, one must be cautious in interpreting the results as impressions are often observed in hospitals’ accounting data.

## Conclusion

Despite the small proportion of ICU beds in relation to the total number of services, the ICU represents a significant cost for the hospital. Furthermore, this study confirms that nursing staff represent a significant proportion of direct ICU costs. The total cost per stay varies by hospital, which can be explained in part by certain medical factors identified in our results. The organisation of ICUs in Belgium could be revised based on our results by implementing different levels of intensive care as in the majority of European countries. The creation of intermediate units could make it possible to avoid hospitalisation and reduce the length of stay in the ICU, thereby reducing hospital costs. In view of the reform of hospital financing that is being prepared in Belgium, based on lump sums per pathology, our results will allow us to deepen our reflections on the appropriateness of financing ICUs by lump seems per DRG.

### Electronic supplementary material

Below is the link to the electronic supplementary material.

Supplementary Material 1

## Data Availability

The dataset used and analysed in this manuscript is available from the corresponding author upon reasonable request.
