# Supplementary material for: Cost comparisons and factors related to cost per stay in intensive care units in Belgium
Source: BMC Health Serv Res. 2023 Sep 13;23:986. doi: 10.1186/s12913-023-09926-2 (PMC10500739; doi:10.1186/s12913-023-09926-2)
Supplement: Supplementary file 1 — Supplementary Material 1 [file 12913_2023_9926_MOESM1_ESM.docx]

Supplementary Material

**Annex 1**: The allocation of direct and indirect costs

| **Direct cost** | **Indirect cost** |
| --- | --- |
| Medical procedures in the intensive care unit (medical doctor costs)  Supplies and materials  Pharmaceutical products  Medical imaging  Laboratory tests  Health professionals costs  Depreciation on equipment | Hotel costs (heating, laundry, food, cleaning, mortuary, etc.)  Administrative costs (nursing management, IT department, telephone service, billing and pricing service, infection prevention and control service, worship, etc.) |
